# Neutralization Dialysis for Phenylalanine and Mineral Salt Separation. Simple Theory and Experiment

**DOI:** 10.3390/membranes9120171

**Published:** 2019-12-10

**Authors:** Anton Kozmai, Elena Goleva, Vera Vasil’eva, Victor Nikonenko, Natalia Pismenskaya

**Affiliations:** 1 Department of Physical Chemistry, Kuban State University, 149 Stavropolskaya Street., 350040 Krasnodar, Russia; v_nikonenko@mail.ru (V.N.); n_pismen@mail.ru (N.P.); 2 Department of Analytical Chemistry, Voronezh State University, 1 Universitetskaya pl., 394018 Voronezh, Russia; vorobjeva_ea@mail.ru (E.G.); viv155@mail.ru (V.V.)

**Keywords:** neutralization dialysis, simulation, experiment, amino acid, mineral salt

## Abstract

A simple non-steady state mathematical model is proposed for the process of purification of an amino acid solution from mineral salts by the method of neutralization dialysis (ND), carried out in a circulating hydrodynamic mode. The model takes into account the characteristics of membranes (thickness, exchange capacity and electric conductivity) and solution (concentration and components nature) as well as the solution flow rate in dialyzer compartments. In contrast to the known models, the new model considers a local change in the ion concentration in membranes and the adjacent diffusion layers. In addition, the model takes into consideration the ability of the amino acid to enter the protonation/deprotonation reactions. A comparison of the results of simulations with experimental data allows us to conclude that the model adequately describes the ND of a strong electrolyte (NaCl) and amino acid (phenylalanine) mixture solutions in the case where the diffusion ability of amino acids in membranes is much less, than mineral salts. An example shows the application of the model to predict the fluxes of salt ions through ion exchange membranes as well as pH of the desalination solution at a higher than in experiments flow rate of solutions in ND dialyzer compartments.

## 1. Introduction

Diffusion (Donnan) dialysis, DD, with the use of ion-exchange membranes is an energy-saving and technically simple process. The elementary unit of the membrane stack for DD consists of two compartments separated by a cation exchange membrane (CEM) or anion exchange membrane (AEM). DD is widely used for purification of the metallurgical industry wastes from heavy metals [1,2]; reduction of acidity of these wastes or reuse of inorganic acid solutions [3,4,5]; recovery of halides or phosphates from these wastes [6,7]; purification of high molecular weight compounds from organic and inorganic electrolytes [8] and separation of amino acids and mineral impurities [9], as well as in various medical applications [10,11,12] and for the precise determination of some substances concentrations in solutions of complex composition [13,14,15].

A number of works are devoted to DD modeling, for example [16,17,18,19,20]. In some of them attempts are made to describe the diffusion and convective transport of molecular and ionic forms of substances through ion exchange membranes (IEM) and adjacent diffusion layers [21], to take into account the facilitated transport of amino acids through CEM being in H^+^ form [22], or, using the enhancement factor for some model parameters, to take into account chemical reaction between components that diffuse through the IEM in opposite directions [23].

Neutralization dialysis (ND) was proposed first by Igawa [24] and has demonstrated a higher efficiency than DD for desalination of multicomponent solutions [25,26], separation of electrolytes (substances that are present in solution in the form of ions and can be transported through IEM) [24], the extraction of weak inorganic electrolytes [27] or carboxylic acids [28], as well as the selective fractionation of various amino acids [29,30] (the substances that participates in protolysis reactions) or mixtures of mineral salt and amino acid. In recent years, new modifications of ND have appeared, for example, capacitive neutralization dialysis, which is used to generate electricity by utilizing acidic and alkaline wastewater using ND techniques in combination with capacitive deionization [31,32].

An elementary cell of the membrane stack for ND consists of three compartments, separated by CEM and AEM. A solution is pumped through the desalination (central) compartment, which contains, for example, a mixture of amino acid with mineral impurities. An inorganic acid is pumped through a compartment adjacent to CEM (acid compartment). An alkali is pumped through a compartment adjacent to AEM (base compartment). Protons are transported from the acid compartment through the CEM to exchange with cations from the desalination compartment. Hydroxyl ions are transported from the base compartment through the AEM to exchange with anions from the desalination compartment. The protons and hydroxyl ions in the desalination compartment react with each other to form water. It has been established that the efficiency of ND demineralization increases with a decrease in the intermembrane distance in the desalination compartment as well as with a decrease in the solution flow rate in the case of the direct-flow hydraulic mode [25]. If a batch hydraulic mode is used, on the contrary, it is necessary to increase the solution flow rate in order to reduce the diffusion boundary layer (DBL) thickness near the membrane surface [33,34]. The degree of extraction (or retention) of substances that enter the protolysis reactions depends on the values of the constants of these reactions and on the concentrations of acid and alkali [27,35] in the corresponding compartments of the ND stack. The amino acid charge can be controlled by adjusting the value of pH in the desalination compartment. If the amino acid is in zwitterionic form, it is not transported through IEMs. Flux through the membrane reaches a maximum if the entire amino acid, for example, glycine [30], is in ionic form. Using a three-compartment ND cell and the principle of controlling the pH values in the desalination compartment, Tsukahara et al. [35] achieved a separation factor of various amino acids equal to 4000.

The modeling of ND is fraught with a number of mathematical difficulties associated with the need to take into account the mutually dependent fluxes of substances through both the CEM and the AEM of the three-compartment elementary unit of membrane stack. Among the models of the ND process, one may emphasis the following ones. Sato et al. [36] proposed a model of continuous ND that describes the process of strong electrolytes desalination and takes into account the diffusion, migration and convective component of ion fluxes. The equations are obtained under the assumption that a quasi-steady state is achieved in the system under study. The numerical solution of these equations made it possible to evaluate quantitatively the influence of the Reynolds numbers and the feeding solution pH upon the average dialysis rates of each of the ions participating in ND process. A similar quasi-steady state model describing the ND kinetics was developed by Denisov et al. [37]. The transport of ions through ideally selective IEMs and DBLs are taken into account in the model. From the calculations using this model, it follows that at high concentrations of acid, mineral salt and alkali in all three compartments of a dialyzer, the pH values in the desalination compartment are determined by the characteristics of the membranes. In contrast, in dilute solutions, mass transfer processes in the diffusion layers control the ion exchange between the compartments. The model predicted the oscillatory nature of the change in pH into the desalination compartment. A periodic increase and decrease in pH into the desalination compartment was experimentally confirmed in [33,34,38]. Models [36,37] were improved in [39], where a batch mode of the ND is considered. This model includes such parameters as IEM exchange capacity, electric conductivity and diffusion layer thicknesses found from the independent experiments. Calculations using this non-steady state model showed that fluctuations in pH and electric conductivity at the initial stages of ND could not be described within the framework of the quasi-steady state model [37]. At the same time, both models give the same results in 20–30 min from the beginning of ND after stationary concentration profiles are formed into the membranes. It should be noted that all these models were developed for the case of a strong electrolyte solution in the desalination compartment.

In this paper, we show that the theoretical approaches developed for ND of strong electrolyte are suitable for predicting the characteristics of the amino acid and mineral salt ND separation process, if the diffusion coefficients of amino acid species are much lower than that of mineral salt. A mixed solution of sodium chloride (NaCl) and phenylalanine (Phe) will be taken as an example. Our relatively simple non-steady state model takes into account the main practically important characteristics of membranes (thickness, exchange capacity and electric conductivity) and solutions (concentrations, diffusion coefficients of components and equilibrium constants of protolysis reactions) as well as the solution flow rate in dialyzer compartments.

In contrast to the known models [35,36], the new model takes into account the local changes in the concentration of ions in membranes and DBLs. In addition, a novelty compared to the model [38] is in accounting the ability of an amino acid in a desalination compartment of the ND system to change the electric charge due to protolysis reactions. We show that the model adequately describes the experimental data obtained for NaCl solution or mixture NaCl + Phe in a desalination compartment. We will also demonstrate the ability of the new model to predict ND process characteristics if experimental data are not available.

## 2. Theoretical

### 2.1. System under Study

**Figure 1:**
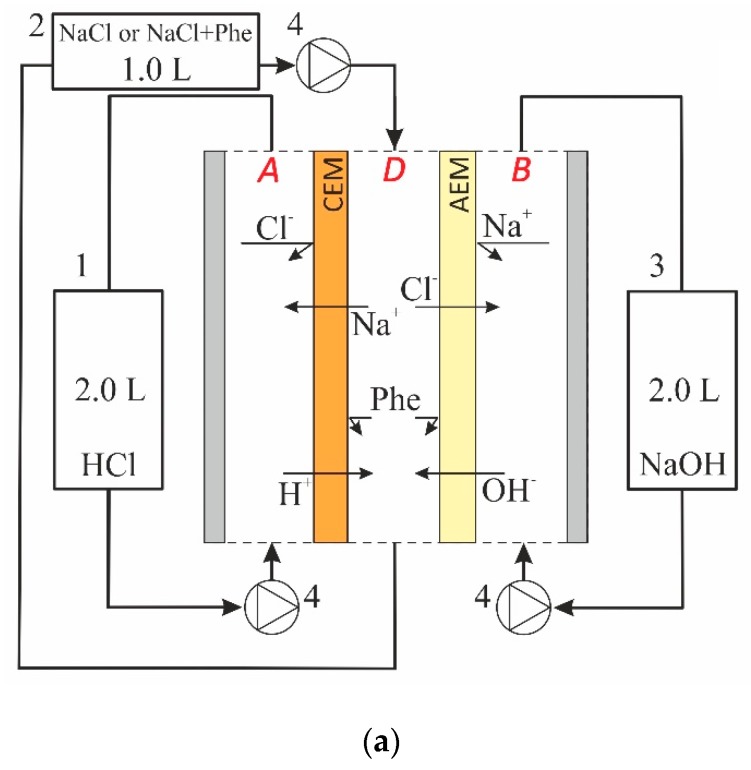

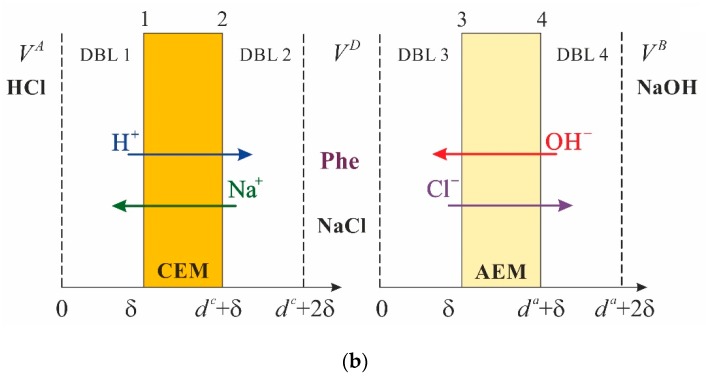
Scheme of the laboratory cell (**a**) and the modeled system geometry (**b**). In (**a**): the acid, saline and alkaline solutions circuits are denoted as 1, 2 and 3, respectively; 4 is the peristaltic pumps. In (**b**) DBL1 and DBL2 are the diffusion boundary layers adjacent to the cation exchange membrane (CEM) from the sides of *A* and *D* compartments, respectively; DBL3 and DBL4 are the diffusion boundary layers adjacent to the anion exchange membrane (AEM) from the sides of *D* and *B* compartments, respectively; the numbers 1, 2, 3 and 4 indicate the interfaces of the CEM and AEM with the corresponding compartments.

The system under study was formed by three compartments: acid (*A*), alkali (*B*) and desalination (*D*), which was separated by CEM and AEM (Figure 1).

Each of the circuits that formed by corresponding compartments together with pipes and intermediate tanks have volumes of *V^A^*, *V^B^* and *V^D^* for the acid, alkali (base) and desalination one, respectively (Figure 1). The acid (HCl), alkali (NaOH) and salt (NaCl) or mixed (NaCl + Phe) solutions circulate through the compartments *A*, *B* and *D*, respectively, and through the intermediate tanks. It is assumed that the thickness ( δ ) of DBLs adjacent to the surfaces of CEM is equal to those adjacent to the surfaces of AEM. The thicknesses of CEM and AEM are denoted as *d^c^* and *d^a^*, respectively. This assumption is reasonable because the hydrodynamic conditions are the same in all the compartments. Besides, by setting the DBL thickness, we implicitly took into account the effect of convection, which depends on the solution velocity, and the ability of the spacer to turbulize the flow. We assumed that the concentrations of all ions practically do not change along compartment *D*. In other words, the ion concentrations are the same at any moment of time both in the compartment volumes and in the corresponding intermediate tanks.

Two cases of ND were considered. In the first case, the desalination of individual NaCl solution was performed. In this case, the problem formulation was the same as in article [39].

In the second case, the mixed NaCl + Phe solution was desalinated. This amino acid was characterized by relatively low diffusion fluxes in thin homogeneous membranes [29]. As it was shown by Vasil’eva et al. [22] the flux of phenylalanine species through the relatively thick MK-40_prof_ membrane (which was used in the current study) is two orders of magnitude lower than that of the salt ions. Thus, for the sake of simplicity, we assumed the constant phenylalanine concentration in the desalination circuit, i.e., there is no transport of Phe species through the membranes and adjacent diffusion layers. At the membrane/solution interfaces a local thermodynamic equilibrium was assumed between exchanging ions. In the desalination solution, we assumed local equilibrium of the protolysis reactions for amino acid and water.

### 2.2. Problem Formulation

The ion transport in membranes and DBLs is described by the following equations [39]:

The Nernst–Planck (N–P) equation

(1) $$J_{j}=-D_{j}\left(\frac{\partial C_{j}}{\partial x}+z_{j}C_{j}\frac{F}{RT}\frac{\partial \varphi }{\partial x}\right),$$

the electroneutrality condition

(2) $$\underset{j}{\Sigma }z_{j}C_{j}=\omega X,$$

the zero current flow condition

(3) $$\underset{j}{\Sigma }z_{j}J_{j}=0,$$

the material balance equation

(4) $$\frac{\partial C_{j}}{\partial t}=-\frac{\partial J_{j}}{\partial x},$$

where $J_{j}$ is the flux density across the membrane; $D_{j}$ is the diffusion coefficient of ion *j*; $C_{j}$ is the concentration; $z_{j}$ is the charge; X is the membrane exchange capacity; ω can take the values of −1, +1 and 0 for an AEM, a CEM and a solution, respectively; *x* is the coordinate perpendicular to the membrane surface; *t* is the time and *R*, *T* and *F* are the gas constant, temperature and Faraday constant, respectively. In the *D* circuit *j* = H^+^, OH^−^, Na^+^, Cl^−^; in the CEM, *j* = H^+^, Na^+^; in the AEM, *j* = OH^−^, Cl^−^.

The following equation describes the equilibrium of the reaction of water autoprotolysis:

(5) $$K_{w}=C_{H}^{}\cdot C_{OH}^{}=10^{-14}.$$

Phenylalanine (2-amino-3-phenylpropanoic acid) is involved in proton transfer reactions with water:

(6) $$Phe^{+}+H_{2}O↔Phe^{\pm }+H_{3}O^{+}.$$

(7) $$Phe^{\pm }+H_{2}O↔Phe^{-}+H_{3}O^{+}\;.$$

Hereinafter the zwitterion ( ${\;}^{+}NH_{3}-CH\left(CH_{2}C_{6}H_{5}\right)-COO^{-}$ ), cation ( ${\;}^{+}NH_{3}-CH\left(CH_{2}C_{6}H_{5}\right)-COOH$ ) and anion ( $NH_{2}-CH\left(CH_{2}C_{6}H_{5}\right)-COO^{-}$ ) of phenylalanine are designated as Phe^±^, Phe^+^ and Phe^−^, correspondently.

The chemical equilibrium constants of reactions (6) and (7) at 25 °C are equal to

(8) $$K_{1}=\frac{\left[Phe^{\pm }\right]\left[H_{3}O^{+}\right]}{\left[Phe^{+}\right]}=6.31\times 10^{-3}\;\mathrm{mol}\;L^{-1},$$

and

(9) $$K_{2}=\frac{\left[Phe^{-}\right]\left[H_{3}O^{+}\right]}{\left[Phe^{\pm }\right]}=4.90\times 10^{-10}\;\mathrm{mol}\;L^{-1},$$

respectively [40]. 

**Figure 2:**
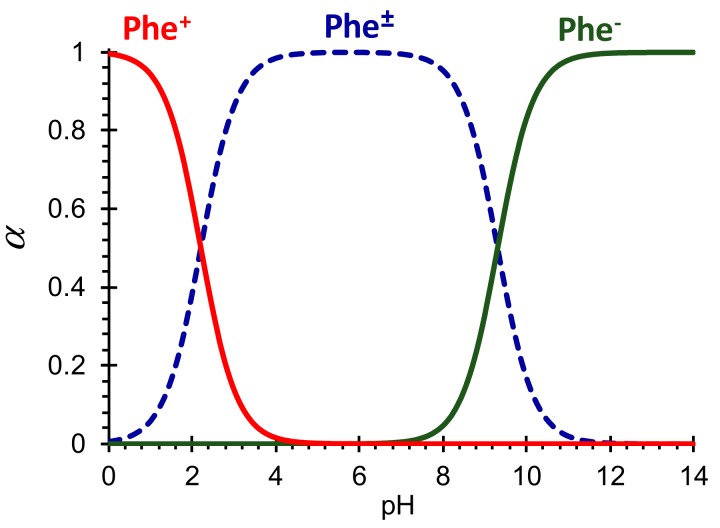
The molar fractions ( α) of phenylalanine species in aqueous solutions as function of pH calculated using Equations (5)–(8), (9).

Thus, depending on the pH of the solution in the desalination compartment, phenylalanine changes its form due to proton transfer reactions (Figure 2), affecting the exchange of salt ions through CEM and AEM. 

Since the phenylalanine ions transport through the membranes is neglected, the changes in concentration of Phe^±^, Phe^+^ and Phe^−^ in the desalination circuit are calculated in accordance with Equations (8) and (9) as follows:

(10) $$C_{Phe^{+}}=\frac{C_{Phe}^{tot}}{\frac{K_{1}K_{2}}{C_{H}^{2}}+\frac{K_{1}}{C_{H}}+1},$$

(11) $$C_{Phe^{-}}=\frac{K_{1}K_{2}C_{Phe^{+}}}{C_{H}^{2}}\;,$$

(12) $$C_{Phe^{\pm }}=\frac{K_{1}C_{Phe^{+}}}{C_{H}}\;,$$

where $C_{Phe}^{tot}=C_{Phe^{\pm }}+C_{Phe^{+}}+C_{Phe^{-}}$ is the total concentration of all the phenylalanine species in the *D* circuit.

The changes in ion concentration in the *A* circuit (Figure 1) depend on the fluxes of H^+^ ( $J_{H}^{\left(1\right)}$ ) and Na^+^ ( $J_{Na}^{\left(1\right)}$ ) ions through the interface 1 of the CEM (Figure 1) as [24]:

(13) $$-\frac{S}{V^{A}}J_{H}^{\left(1\right)}=\frac{\partial C_{H}^{A}}{\partial t},$$

(14) $$-\frac{S}{V^{A}}J_{Na}^{\left(1\right)}=\frac{\partial C_{Na}^{A}}{\partial t}\;,$$

where *S* is the active membrane surface area.

Note that if the direction of flux coincides with the positive direction of axis *x*, this flux is considered positive.

The changes in ion concentration in the *B* circuit (Figure 1) are associated with the fluxes of OH^−^ ( $J_{OH}^{\left(4\right)}$ ) and Cl^−^ ( $J_{Cl}^{\left(4\right)}$ ) ions through the interface 4 of the AEM:

(15) $$\frac{S}{V^{B}}J_{OH}^{\left(4\right)}=\frac{\partial C_{OH}^{B}}{\partial t}.$$

(16) $$\frac{S}{V^{B}}J_{Cl}^{\left(4\right)}=\frac{\partial C_{Cl}^{B}}{\partial t}\;.$$

The fluxes through boundaries 2 and 3 are associated with the changes in the concentration of saline solution in the *D* circuit (Figure 1):

(17) $$-\frac{S}{V^{D}}J_{Cl}^{\left(3\right)}=\frac{\partial C_{Cl}^{D}}{\partial t}.$$

(18) $$\frac{S}{V^{D}}\left(J_{H}^{\left(2\right)}+J_{OH}^{\left(3\right)}\right)=\frac{\partial }{\partial t}\left(C_{H}^{D}-C_{OH}^{D}\right)\;.$$

The Na^+^ ions concentration in the *D* circuit is calculated using the electroneutrality condition (2), where the presence of Phe^+^ and Phe^−^ ions is taken into account:

(19) $$C_{Na}^{D}=C_{Phe^{-}}+C_{Cl}^{D}+C_{OH}^{D}-C_{H}^{D}-C_{Phe^{+}}.$$

At the membrane/solution interfaces, the ion-exchange equilibrium as well as the continuity of fluxes of ions are assumed.

The membrane/solution interfaces are characterized by the local equilibrium described by Nikolskii equations [37]:

(20) $$K_{}^{c}=C_{H}^{c}C_{Na}/C_{Na}^{c}C_{H},$$

(21) $$K_{}^{a}=C_{OH}^{a}C_{Cl}/C_{Cl}^{a}C_{OH}\;,$$

where $C_{j}^{c}$ and $C_{j}^{a}$ are the interfacial concentrations of ions *j* into the CEM and AEM, respectively; $C_{j}$ is the interfacial concentration of these ions in the solution; $K^{c}$ is the equilibrium coefficient for H^+^/Na^+^ exchange (interfaces 1 and 2) and $K^{a}$ is the equilibrium coefficient for OH^−^/Cl^−^ exchange (interfaces 3 and 4).

The condition of the flux continuity at the interfaces 1 and 2 reads as:

(22) $${\left(J_{j}\right)}_{\begin{array}{l} x=\delta \\ x=d^{c}+\delta \end{array}}={\left(J_{j}^{c}\right)}_{\begin{array}{l} x=\delta \\ x=d^{c}+\delta \end{array}}.$$

The concentration continuity is assumed at the DBL/solution bulk boundaries:

(23) $${\left(C_{j}\right)}_{\begin{array}{l} x=0\\ x=d^{c}+2\delta \end{array}}=C_{j}^{A,D},$$

where $C_{j}^{A,D}$ is the concentration of ion *j* in circuits *A* or *D*. 

The boundary conditions at the AEM side are similar.

The electric potential can be expressed from Equations (1)–(3). In the case of CEM it reads as:

(24) $$\frac{\partial \varphi^{c}}{\partial x}=\frac{RT}{F}\cdot \frac{\left(D_{Na}^{c}-D_{H}^{c}\right)\frac{\partial C_{H}^{c}}{\partial x}}{\left(D_{H}^{c}-D_{Na}^{c}\right)C_{H}^{c}+D_{Na}^{c}X^{c}},$$

and in the case of AEM:

(25) $$\frac{\partial \varphi^{a}}{\partial x}=\frac{RT}{F}\cdot \frac{\left(D_{OH}^{a}-D_{Cl}^{a}\right)\frac{\partial C_{OH}^{a}}{\partial x}}{\left(D_{OH}^{a}-D_{Cl}^{a}\right)C_{OH}^{a}+D_{Cl}^{a}X^{a}}.$$

In a similar way, the equation for the potential in the DBL1 can be derived:

(26) $$\frac{\partial \varphi^{DBL1}}{\partial x}=\frac{RT}{F}\frac{\left(D_{Cl}^{}-D_{H}^{}\right)\frac{\partial C_{H}^{}}{\partial x}+\left(D_{Cl}^{}-D_{Na}^{}\right)\frac{\partial C_{Na}^{}}{\partial x}}{\left(D_{Cl}^{}+D_{H}^{}\right)C_{H}^{}+\left(D_{Cl}^{}+D_{Na}^{}\right)C_{Na}^{}},$$

and in the DBL4:

(27) $$\frac{\partial \varphi^{DBL4}}{\partial x}=\frac{RT}{F}\frac{\left(D_{OH}^{}-D_{Na}^{}\right)\frac{\partial C_{OH}^{}}{\partial x}+\left(D_{Cl}^{}-D_{Na}^{}\right)\frac{\partial C_{Cl}^{}}{\partial x}}{\left(D_{OH}^{}+D_{Na}^{}\right)C_{OH}^{}+\left(D_{Cl}^{}+D_{Na}^{}\right)C_{Cl}^{}}.$$

The equation for the potential in the DBL2 reads as [39]:

(28) $$\frac{\partial \varphi^{DBL2}}{\partial x}=\frac{RT}{F}\cdot \frac{\left[D_{Cl}^{}-D_{H}^{}+\frac{K_{w}}{{\left(C_{H}^{}\right)}^{2}}\left(D_{Cl}^{}-D_{OH}^{}\right)\right]\frac{\partial C_{H}^{}}{\partial x}+\left(D_{Cl}^{}-D_{Na}^{}\right)\frac{\partial C_{Na}^{}}{\partial x}}{\left(D_{OH}^{}-D_{Cl}^{}\right)\frac{K_{w}}{C_{H}^{}}+\left(D_{H}^{}+D_{Cl}^{}\right)C_{H}^{}+\left(D_{Na}^{}+D_{Cl}^{}\right)C_{Na}^{}},$$

and that in the DBL3:

(29) $$\frac{\partial \varphi^{DBL3}}{\partial x}=\frac{RT}{F}\cdot \frac{\left[D_{OH}^{}-D_{Na}^{}+\frac{K_{w}}{{\left(C_{OH}^{}\right)}^{2}}\left(D_{H}^{}-D_{Na}^{}\right)\right]\frac{\partial C_{OH}^{}}{\partial x}+\left(D_{Cl}^{}-D_{Na}^{}\right)\frac{\partial C_{Cl}^{}}{\partial x}}{\left(D_{H}^{}-D_{Na}^{}\right)\frac{K_{w}}{C_{OH}^{}}+\left(D_{OH}^{}+D_{Na}^{}\right)C_{OH}^{}+\left(D_{Na}^{}+D_{Cl}^{}\right)C_{Cl}^{}}.$$

Further, the problem was solved numerically after the substitution of Equations (24)–(29) into the Nernst–Planck equations (1) taking into account boundary conditions (22) and (23) and Equations (4) and (10)–(12).

The uniform distribution of concentrations in the DBLs (which are equal to the concentration of initial feed solution) was assumed as the initial conditions for the problem described above (at the beginning of the ND process, *t* = 0).

## 3. Experiment

The ND elementary cell (Figure 1) contained heterogeneous cation (MK-40_prof_) and anion-exchange (MA-40_prof_) membranes with a profiled surface. Detailed characteristics of the profile on the surface of studied membranes are presented in [22]. The basic characteristics of these membranes are presented in Table 1. The JSC Innovative Enterprise “Membrane Technology” (Russia) manufactured profiled membranes from flat sheets, which are produced by “Shchekinoazot” (Russia).

The MK-40_prof_ and MA-40_prof_ membranes were chosen under the assumption that the relatively large thickness of these membranes and their aromatic polymer matrix will contribute to the retention of aromatic phenylalanine in the desalination compartment while maintaining high fluxes of salt ions through CEM and AEM.

The active area of membranes was 4.2 × 1.7 cm². The intermembrane distance was 0.6 cm; it did not contain spacer in order to have a possibility to calculate the diffusion boundary layer thickness using the convective-diffusion model [22].

The studied solution circulated through the *D* compartment; the acid and alkali solutions circulated through the *A* and *B* compartments, correspondingly (Figure 1). The initial concentration of salt and amino acid in individual (NaCl) solution and the equimolar mixture NaCl + Phe in the desalination circuit (Figure 1) was 0.025 mol L^−1^. Both the HCl and NaOH solutions had the concentration of 0.3 mol L^−1^. The choice of these concentrations was based on the analysis of phenylalanine and sodium chloride separation efficiency by diffusion dialysis method [22]. The volume of solution in *D* circuit was 1 L, and that in *A* and *B* circuits was 2 L. The ND process was carried out in a circulating (batch) hydrodynamic mode. The solution volumetric flow velocity in each of the compartments was equal to 12 mL min^−1^ (the corresponding linear flow velocity was 0.2 cm s^−1^). The calculated DBL thickness in this case was about 400 μm (Table A1, Appendix A). A higher value of the DBL thickness (than in real electrodialysis) was chosen in order to partially level the internal diffusion kinetics of the ND process, which could be due to the relatively large thickness of the membranes under study. The solutions were temperature-stabilized at 25 °C.

Before the experiment, the membranes underwent salt pretreatment in accordance with the procedure described in [42], and then they are converted to the form of H^+^ (CEM) and OH^−^ (AEM) ions. All solutions were prepared using distilled water and reagents of analytic grade. The pH of the feed solutions in the experiments was equal to 5.90 ± 0.05. This value was close to the isoelectric point *pI* = (*pK*_1_ + *pK*_2_)/2 = 5.76 [43]. As the estimates (made with the use of Equations (8) and (9) at the presented above values of *K*_1_ and *K*_2_) [22] show, the zwitterion mole fraction in the model solutions varied in the range from 99.9% to 99.8% where the pH changed between 5.85 and 5.95, respectively. Thus, the phenylalanine almost completely was presented in the form of zwitterion in the initial solution. 

Absorption spectroscopy at a wavelength of 257 nm was applied to determine the concentration of phenylalanine in the solution. The concentration of Na^+^ ions was determined using the emission flame photometry.

## 4. Parameters of the Model

A list that sums up all the model parameters is presented in Appendix A, Table A1.

Three groups of the input model parameters characterizing the membrane were: thermodynamic, structural and kinetic. The parameters characterizing solutions were the pH and the concentration of electrolytes. In addition, the model contained the parameters characterizing the ND setup.

The thermodynamic parameters were the Nikolskii ( K; Equations (20) and (21)) equilibrium constants; the equilibrium constants for reactions (6) and (7) ( $K_{i}$ ).

The membrane was described by the ion-exchange capacity ( X ) and thickness ( d ).

Kinetic parameters were the ion diffusion coefficients in the membrane and the solution ( $D_{j}$ ).

The ND setup was characterized by the volume of solutions in the *A*, *B* and *D* circuits ( $V^{A}$, $V^{B}$ and $V^{D}$ ) and by membrane active surface area ( S ).

The output parameters of the model included the Na^+^, Cl^−^, H^+^ and OH^−^ ions concentrations and fluxes as functions of time and the coordinate (normal to the membrane surface) in the membranes and solutions, as well as concentrations of Phe^±^, Phe^+^ and Phe^−^ in the *D* circuit as functions of time.

The input parameters that were not taken from the literature were determined as follows.

(1) The values of the Nikolskii constants were taken equal to 1. For the sake of simplicity, we assumed that there were no preferential counterions in their sorption by membranes.

(2) The counterions diffusion coefficients in CEM and AEM were determined by using the relationship $D_{j}^{k}=\kappa^{k}RT/X^{k}F^{2}$ [34]. In this equation, the membrane conductivity, $\kappa^{k}$, and the exchange capacity, $X^{k}$, were known (Table 1). Here *k* = c for the CEM and *k* = a for the AEM.

(3) The membrane exchange capacity was determined by the method of reverse acid-base titration [41].

(4) The thickness of studied membranes (in the swollen state) was measured using an electronic micrometer.

The diffusion boundary layer thickness (δ) was the fitting parameter. It was assumed that δ was the same from the side of *A*, *B* and *D* compartments.

## 5. Results and Discussion

**Figure 3:**
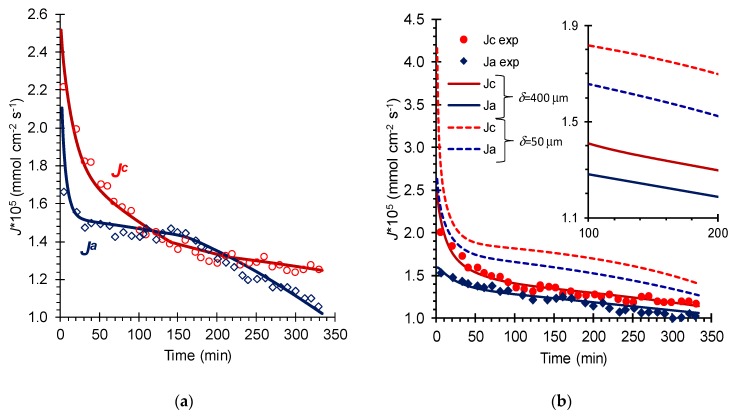
Time dependencies of Na^+^ ions flux (*J^c^*) across the CEM and Cl^−^ ions flux (*J^a^*) across the AEM from the *D* compartment into *A* and *B* compartments, respectively, in the course of neutralization dialysis (ND) of individual NaCl solution (**a**) and mixed equimolar NaCl + Phe solution (**b**). Markers indicate the experimental data. Lines indicate the results of simulations carried out for *δ* = 400 μm (solid lines) and for *δ* = 50 μm (dashed lines). Other parameters for calculations correspond to conditions of the experiment (Table A1, Appendix A).

**Figure 4:**
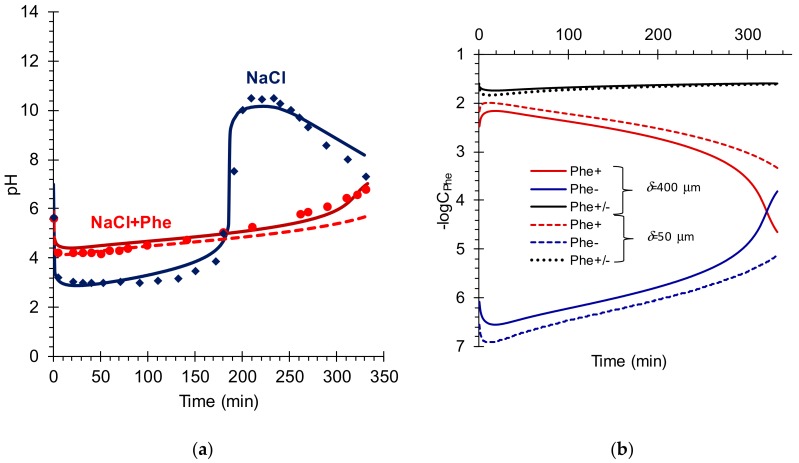
The pH of individual NaCl and mixed NaCl + Phe solutions (**a**), and concentrations of phenylalanine species (mixed solution) in the *D* circuit vs. time of ND process (**b**). Markers indicate the experimental data. Lines indicate the results of simulations carried out for *δ* = 400 μm (solid lines) and for *δ* = 50 μm (dashed lines). Other parameters for calculations correspond to conditions of the experiment (Table A1, Appendix A).

The kinetic dependencies of the calculated and experimental salt ions fluxes from the *D* compartment to the acid, *A*, and base, *B*, compartments are presented in Figure 3. The kinetic dependencies of pH of the saline solution, as well as the concentrations of phenylalanine species (the case of mixed NaCl + Phe solution) are presented in Figure 4. 

As Figure 3 and Figure 4 show, the results of simulations by the proposed model and experimental data were in a good agreement. As expected, the flux of Na^+^ ions through CEM exceeded the flux of Cl^−^ ions through AEM (Figure 3a) at the beginning of the ND process. Then they changed places. At the last stage of the ND process, the transfer of cations through CEM again dominated the transfer of anions through AEM. The changes in fluxes were accompanied by significant fluctuations in the pH of solution in the *D* compartment (Figure 4a). It means that a higher exchange rate across the CEM than that across the AEM at the beginning of the ND process (Figure 3a) led to a decrease in the pH of the desalinated solution (Figure 4a). In the next 50 min of the ND process, there was an increase in the concentration of H^+^ ions and a decrease in the concentration of Na^+^ ions in the *D* circuit. The concentrations gradients of cations in the CEM decreased, causing a decrease in cation flux (*J^c^*) through this membrane. Next, this scenario was repeated.

A comparison of the experimental data and the results of simulation, as well as analysis of the literature data [39] allowed us to propose the following explanation for pH fluctuations in the case of a strong electrolyte (NaCl) solution desalination. 

The exchange of anions across the AEM occurred simultaneously with the exchange of cations across the CEM. However, the exchange rate across the AEM was lower due to the lower diffusion coefficient of OH^−^ ions in the AEM than H^+^ in the CEM. It is known that sulfo groups, which are fixed groups in the studied CEM, provide a high proton transfer rate [41]. This rate is noticeably higher than that for the hydroxyl ion with the participation of weakly basic fixed groups [41] in studied AEM. This is evidenced, for example, by the specific electric conductivity of MK-40 and MA-40 membranes in 0.1 M HCl and NaOH solutions, respectively. The values of this conductivity are 36 mS cm^−1^ [44] and 6.4 mS cm^−1^ [45], respectively. For this reason, there was a less dramatic decrease in the exchange rate (flux) across the AEM (compared to that across the CEM; Figure 3a). In addition, the achieved low pH value of the desalinated solution stimulated the increase in *J^a^*. Due to the delay in the formation of concentration profiles in the AEM and adjacent diffusion layers, the moment where the exchange rate across the AEM decreases shifted in time.

In the case of a mixed NaCl + Phe solution, the behavior of the fluxes of cations and anions in the membrane system (Figure 3b), as well as the change in pH in the desalination compartment (Figure 4a) at the beginning of ND, was similar to that observed in the case of an individual solution. However, the course of ND process was accompanied by a monotonous decrease in *J^c^*, *J^a^* (Figure 3b) and a slow increase in the value of the solution pH in the desalination compartment (Figure 4a). The increase in pH led to a slight decrease in the concentration of phenylalanine cations and an increase in concentration of its anions (Figure 4b). The concentrations of these ions turned out to be two to five orders of magnitude lower than the concentration of the zwitterionic form (Figure 4b). Therefore, the loss of amino acid in the process of ND did not exceed 1% if we assumed that all of charged Phe forms would be transported through the IEM. Similar values of amino acid loss were measured by us experimentally. This justified the assumption made in the simulation of ND that the concentration of Phe in the *D* circuit remained unchanged.

Apparently, the phenomena that occur in the case of NaCl solution and mixed NaCl + Phe solutions in ND membrane stack were similar. However, buffer capacity of the amino acid solution due to the protonation/deprotonation reactions (Equations (6) and (7)) prevented fluctuations in the pH values in the desalination compartment, and this led to a smoothing of the kinetic dependences of the cations and anions fluxes through corresponding membranes.

The developed model allowed simulating salt ions fluxes in the course of ND process in more favorable (for example, hydrodynamic) conditions than those applied in our laboratory experiments. As a rule, the diffusion layer thickness can be reduced with 1) the decrease in the distance between neighboring membranes, 2) the increase in solution flow velocity [34,36] and 3) the use of spacers with high capability of flow turbulization [26,33,46]. Let us consider one more DBL thickness (except that estimated for the experimental conditions), which was close to 50 μm. This thickness is considered preferred for industrial dialysis processes [29]. The results of these simulations for a mixed NaCl + Phe solution are presented in Figure 3b and Figure 4. Dashed or dotted lines indicate the calculated curves. As expected, a decrease in the DBL thickness led to a noticeable increase in *J^c^* and *J^a^* (Figure 3b). These data were in good agreement with published data, for example [34]. As simulations show, the decrease in DBL thickness by eight times led to an increase in cations flux approximately by 1.7 times in the beginning of the ND process (Figure 3b). In a quasi-steady state (after about 100 min) the desalination rate was about two times higher than that for the DBL thickness equal to 400 microns. 

Note, that in the case of *δ* = 50 μm pH of the desalinated solution decreased more intensively in the beginning of the ND process, increased more slowly and reached the final value 5.7 vs. pH = 7.3 in the case where DBL thickness was equal to 400 microns (Figure 4a). In this case, the expected loss of amino acid in the *D* circuit due to transport of charged Phe species through the membranes were equal about to 2 % vs. 1% determined at the value of *δ* = 400 μm. An increase in amino acid loss in the case where *δ* = 50 μm was apparently associated with a greater proton flux through the CEM + DBL2, which caused a stronger decrease in the saline solution pH and the formation of greater mole fraction of Phe^+^ cations (Figure 4b).

**Figure 5:**
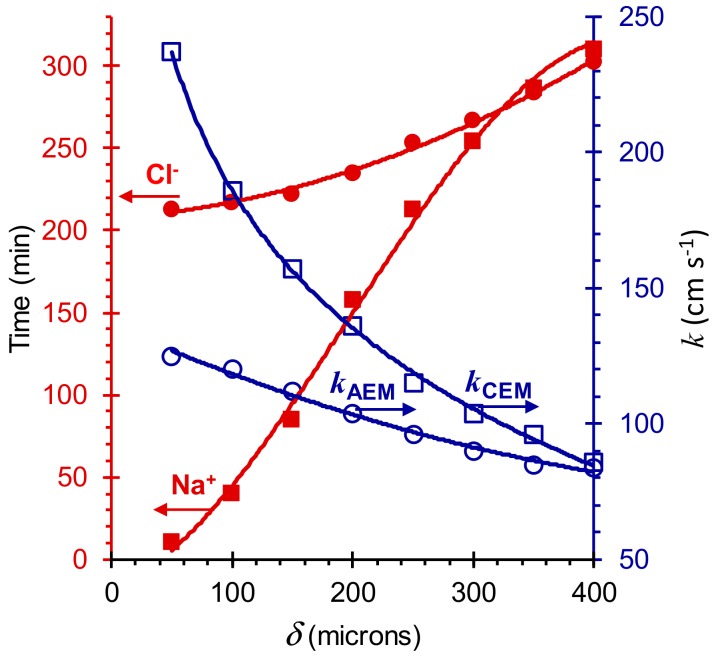
The time required to reduce the concentration of Na^+^ and Cl^−^ ions in the desalination circuit by 50%, as well as the ion transfer coefficients for CEM and AEM vs. DBL thickness. Calculations are made for ND desalination of a mixed NaCl + Phe solutions. Parameters for calculations are presented in Table A1, Appendix A.

Figure 5 shows the calculation results of the time required to reduce the concentration of Na^+^ and Cl^−^ ions in the desalination circuit by two times, as well as the ion transfer coefficients for CEM and AEM (*k_i_ = J_i_/C_i_*). The simulations were carried out for DBL thicknesses from 50 to 400 μm. It could be seen that a decrease in DBL thickness by eight times led to a reduction in desalination time by almost 30 times in the case of Na^+^ ions and only 1.5 times in the case of Cl^−^ ions. The difference between the mass transfer coefficients of Na^+^ ions through CEM and Cl^−^ ions through AEM and, accordingly, the concentrations of these ions in the desalination circuit decreased with increasing DBL thickness. This is explained by the fact that in the case of a thick DBL, the characteristics of the ND process are mainly determined by the external diffusion kinetics. On the contrary, if the DBL thickness is small, the main role is played by the characteristics of the membranes. In this particular case, the transport characteristics of the AEM are much worse than the characteristics of the CEM. By varying the parameters of the membranes in the calculations, it is possible to find the optimal combination of their characteristics, which will ensure the same rates of extraction of salt cations and anions from the desalination circuit of the dialyzer. We planned to do such calculations and their experimental verification in the future.

## 6. Conclusions

A relatively simple non-steady state model that describes the desalination of salt and mixed (salt + amino acid) solutions during neutralization dialysis (ND) was proposed. The model took into account the characteristics of membranes that were important for practice (thickness, exchange capacity and electric conductivity); the concentration of electrolytes and the ability of an amino acid to change electric charge depending on the pH of the medium due to protolysis reaction. In addition, it took into account implicitly the solution flow rate in the dialyzer compartments by the diffusion boundary layer thickness.

The validity of the model was confirmed experimentally using a laboratory dialysis cell formed by profiled heterogeneous cation and anion exchange membranes. Non steady-state ND process carried out using individual salt solution (NaCl) or equimolar mixture of NaCl and phenylalanine solutions (mixed solution).

The model adequately described the time dependences of the salt anions fluxes through anion exchange membrane (AEM) and cations fluxes through cation exchange membrane (CEM) that separates desalination, acid and base (alkali) compartments of the dialyzer. 

The analysis of theoretical and experimental results shows that in the case of demineralization of strong electrolyte (NaCl) solution, the pH fluctuations in the desalination compartment were caused by the following mechanism. The dominance of the exchange rate of cations through CEM caused a significant decrease in pH in the desalination circuit. This decrease in pH entailed an increase in exchange rate of anions through AEM, however, this process proceeded with a certain delay with respect to the pH shift. The dominance of the exchange rate of anions through AEM caused the pH of the solution in the desalination circuit to change in the opposite direction, etc.

The participation of phenylalanine in protonation/deprotonation reactions with protons and hydroxyl ions, which entered the desalination compartment from the acid and alkaline compartments, respectively, imparted a buffering property to the mixed solution of sodium chloride and amino acid. This allowed for avoiding fluctuations in the pH of the solution in the desalination circuit as well as the fluctuations of the salt ion fluxes through the AEM and CEM. 

The model was suitable for predicting the behavior of the studied membrane system if the diffusion coefficients of amino acid and salt ions in ion-exchange membranes differed by two or more orders of magnitude. In addition to phenylalanine, it can be tryptophan, tyrosine, histidine or other high molecular weight substances that are involved in protolysis reactions.

## Figures and Tables

**Table 1:** Some physicochemical characteristics of studied membranes (experimental data).

Membranes	MK-40_prof_	MA-40_prof_
Maximum ^1^ thickness in swollen state (cm)	0.065 ± 0.0005 [22]	0.059 ± 0.0005
Minimum ^2^ thickness in swollen state (cm)	0.030 ± 0.0005	0.030 ± 0.0005
Water content (wt %)	42 ± 1 [22]	44 ± 2
Ion-exchange capacity (meq cm^−3^ wet membrane)	1.7 ± 0.1 [22]	2.4 ± 0.1
Electric conductivity in 0.1 M NaCl (S m^−1^)	0.58 [22]	0.39 [41]

^1^ The membrane thickness between the smooth surface and the top of the profile on the profiled surface. ^2^ The membrane thickness between the smooth surface and the bottom of the profile on the profiled surface.

## Appendix A

**Table A1:** Parameters of the model.

Parameter	Value	Description
$D_{H}^{c}$	2.7 × 10^−6^ cm^2^ s^−1^	H^+^ and Na^+^ ions diffusion coefficients in the MK-40_prof_
$D_{Na}^{c}$	7.88 × 10^−7^ cm^2^ s^−1^	
$D_{OH}^{a}$	9.6 × 10^−7^ cm^2^ s^−1^	OH^−^ and Cl^−^ ions diffusion coefficients in the MA-40_prof_
$D_{Cl}^{a}$	3.03 × 10^−7^ cm^2^ s^−1^	
$d^{a}$ and $d^{c}$	590 and 650 µm	Thickness of the MA-40_prof_ and MK-40_prof_, respectively
$D_{H}^{0}$	9.3 × 10^−5^ cm^2^ s^−1^	H^+^, OH^−^, Na^+^ and Cl^−^ ions diffusion coefficients in solution at infinite dilution
$D_{OH}^{0}$	5.3 × 10^−5^ cm^2^ s^−1^	
$D_{Na}^{0}$	1.33 × 10^−5^ cm^2^ s^−1^	
$D_{Cl}^{0}$	2.03 × 10^−5^ cm^2^ s^−1^	
$C_{H}^{A}$	0.3 mmol cm^−3^	H^+^ and OH^−^ ions initial concentrations in the *A* and *B* circuits, respectively
$C_{OH}^{B}$	0.3 mmol cm^−3^	
$C_{Na}^{D}$	0.025 mmol cm^−3^	Na^+^, Cl^−^, H^+^ and OH^−^ ions initial concentrations in the *D* circuit
$C_{Cl}^{D}$	0.025 mmol cm^−3^	
$C_{H}^{D}$	10^−5.9^ mmol cm^−3^	
$C_{OH}^{D}$	10^−8.1^ mmol cm^−3^	
$C_{Phe}^{D}$	0.025 mmol cm^−3^	Initial concentration of phenylalanine in the *D* circuit
$X^{c}$	1.7 mmol cm^−3^	Ion-exchange capacity of the MK-40_prof_ and MA-40_prof_
$X^{a}$	2.4 mmol cm^−3^	
$K^{c,a}$	1.0	Nikolskii equilibrium constant (upper indexes “c” and “a” denote the MK-40_prof_ and MA-40_prof_ membranes, respectively)
$K_{i}$ (*i* = 1,2)	$K_{1}$*=* 6.31 × 10^−3^ mol L^−1^ $K_{2}$ *=* 4.90 × 10^−10^ mol L^−1^	Equilibrium constants for the phenylalanine protonation/deprotonation chemical reactions in Equations (6) and (7) denoted by ( $K_{1}$ ) and ( $K_{2}$ ), respectively
$V^{A}$	2000 cm^3^	Solution volumes in *A*, *B* and *D* circuits
$V^{B}$	2000 cm^3^	
$V^{D}$	1000 cm^3^	
*S*	7.14 cm^2^	Working surface area of membrane
*F*	96.485 C mmol^−1^	Faraday constant
*R*	8.314 × 10^−3^ J mmol^−1^ K^−1^	Gas constant
*T*	298 K	Absolute temperature

## References

[1] Amrane C., Lalmi A., Bouhidel K.E. (2017). Coupling diffusion dialysis with precipitation-cementation to separate and recover nitric acid, Cu^++^, Zn^++^ and Pb^++^ from the wastewater of a brass pickling bath. Int. J. Glob. Warm..

[2] Kerr C. (2004). Sustainable technologies for the regeneration of acidic tin stripping solutions used in PCB fabrication. Circuit World.

[3] Luo J., Wu C., Xu T., Wu Y. (2011). Diffusion dialysis-concept, principle and applications. J. Membr. Sci..

[4] Janiszewska M., Arguillarena A., Wajs M., Staszak K., Regel-Rosocka M. (2019). Application of diffusion dialysis for reduction of acidity of real pregnant leach solutions containing Ni and Co ions. Sep. Sci. Technol..

[5] Khan M.I., Mondal A.N., Cheng C., Pan J., Emmanuel K., Wu L., Xu T. (2016). Porous BPPO-based membranes modified by aromatic amine for acid recovery. Sep. Purif. Technol..

[6] Xiao H.-F., Chen Q., Cheng H., Li X.M., Qin W.M., Chen B.S., Xiao D., Zhang W.M. (2017). Selective removal of halides from spent zinc sulfate electrolyte by diffusion dialysis. J. Membr. Sci..

[7] Noubli A., Akretche D.E., Crespo J.G., Velizarov S. (2020). Complementary membrane-based processes for recovery and preconcentration of phosphate from industrial wastewater. Sep. Purif. Technol..

[8] Skopinska-Wisniewska J., Olszewski K., Bajek A., Rynkiewicz A., Sionkowska A. (2014). Dialysis as a method of obtaining neutral collagen gels. Mater. Sci. Eng. C.

[9] Vasil’eva V.I., Goleva E.A. (2013). Selective separation of sodium ions from a mixture with phenylalanine by Donnan dialysis with a profiled sulfogroup cation exchange membrane. Rus. J. Phys. Chem. A.

[10] Stamatialis D.F., Papenburg B.J., Gironés M., Saiful S., Bettahalli S.N.M., Schmitmeier S., Wessling M. (2008). Medical applications of membranes: Drug delivery, artificial organs and tissue engineering. J. Membr. Sci..

[11] Tijink M.S.L., Wester M., Sun J., Saris A., Bolhuis-Versteeg L.A.M., Saiful S., Joles J.A., Borneman Z., Wessling M., Stamatialis D.F. (2012). A novel approach for blood purification: Mixed-matrix membranes combining diffusion and adsorption in one step. Acta Biomater..

[12] Yamaguchi N., Miyamoto K., Murata T., Ishikawa E., Horiuchi T. (2016). Newly developed neutralized pH icodextrin dialysis fluid: nonclinical evaluation. Artif. Organs.

[13] Stancheva K.A. (2008). Applications of dialysis. Oxid. Commun..

[14] Radke W. (2016). Consequences of on-line dialysis on polyelectrolyte molar masses determined by size-exclusion chromatography with light scattering detection. J. Sep. Sci..

[15] Huang R.L., Tan Z.L., Xing T.X., Pan Y.F., Li T.J. (2000). An in vitro method for the estimation of ileal crude protein and amino acids digestibility using the dialysis tubing for pig feedstuffs. Anim. Feed Sci. Tech..

[16] Wijmans J.G., Baker R.W. (1995). The solution-diffusion model: a review. J. Membr. Sci..

[17] Ring S., Hasson D., Shemer H., Semiat R. (2015). Simple modeling of Donnan separation processes. J. Membr. Sci..

[18] Agarwal C., Goswami A. (2016). Nernst Planck approach based on non-steady state flux for transport in a Donnan dialysis process. J. Membr. Sci..

[19] Szczepański P., Szczepańska G. (2017). Donnan dialysis—A new predictive model for non−steady state transport. J. Membr. Sci..

[20] Szczepański P. (2018). Chemometric method for Donnan dialysis physicochemical model simplification. Prediction of: Transport, recovery, concentration, and desalination efficiency. Desalination.

[21] Prado-Rubio O.A., Møllerhøj M., Jørgensen S.B., Jonsson G. (2010). Modeling Donnan dialysis separation for carboxylic anion recovery. Comput. Chem. Eng..

[22] Vasil’eva V., Goleva E., Pismenskaya N., Kozmai A., Nikonenko V. (2019). Effect of surface profiling of a cation-exchange membrane on the phenylalanine and NaCl separation performances in diffusion dialysis. Sep. Purif. Technol..

[23] Štěpánek V., Palatý Z., Bendová H. (2015). Numerical analysis of dialysis with chemical reaction at steady state. Irreversible second-order reaction. Chem. Eng. Process..

[24] Igawa M., Echizenya K., Hayashita T., Seno M. (1987). Neutralization dialysis for deionization. Bull. Chem. Soc. Jpn..

[25] Igawa M., Mikami K., Okochi H. (2003). Transport characteristics of neutralization dialysis and desalination of tap water. Bull. Chem. Soc. Jpn..

[26] Bleha M., Tishchenko G.A. (1992). Neutralization dialysis for desalination. J. Membr. Sci..

[27] Tanabe H., Okochi H., Igawa M. (1995). Separation of weak acids and bases by neutralization dialysis. Ind. Eng. Chem. Res..

[28] Zheleznov A., Windmöller D., Körner S., Böddeker K.W. (1998). Dialytic transport of carboxylic acids through an anion exchange membrane. J. Membr. Sci..

[29] Ueno K., Doi T., Nanzai B., Igawa M. (2017). Selective transport of neutral amino acids across a double-membrane system comprising cation and anion exchange membranes. J. Membr. Sci..

[30] Wang G., Tanabe H., Igawa M. (1995). Transport of glycine by neutralization dialysis. J. Membr. Sci..

[31] Wang M., Hou S., Liu Y., Xu X., Lu T., Zhao R., Pan L. (2016). Capacitive neutralization deionization with flow electrodes. Electrochim. Acta.

[32] Liu Y., Zhang Y., Ou-Yang W., Bastos Sales B., Sun Z., Liu F., Zhao R. (2017). Capacitive neutralization dialysis for direct energy generation. Envir. Sci. Tech..

[33] Chérif M., Mkacher I., Ghalloussi R., Chaabane L., Ben Salah A., Walha K., Dammak L., Grande D. (2015). Experimental investigation of neutralization dialysis in three-compartment membrane stack. Desalin. Water Treat..

[34] Chérif M., Mkacher I., Dammak L., Ben Salah A., Walha K., Grande D., Nikonenko V. (2015). Water desalination by neutralization dialysis with ion-exchange membranes: Flow rate and acid/alkali concentration effects. Desalination.

[35] Tsukahara S., Nanzai B., Igawa M. (2013). Selective transport of amino acids across a double membrane system composed of a cation- and an anion-exchange membrane. J. Membr. Sci..

[36] Sato K., Yonemoto T., Tadaki T. (1993). Modeling of ionic transport in neutralization dialytic deionization. J. Chem. Eng. Jpn..

[37] Denisov G.A., Tishchenko G.A., Bleha M., Shataeva L.K. (1995). Theoretical analysis of neutralization dialysis in the three-compartment membrane cell. J. Membr. Sci..

[38] Chérif M., Korchane S., Chaabane L., Dammak L., Ben Salah A., Walha K., Kozmai A. (2017). Reconstituted and brackish waters desalination by neutralization dialysis process with ion-exchange membranes. Desalin. Water Treat..

[39] Kozmai A., Chérif M., Dammak L., Bdiri M., Larchet C., Nikonenko V. (2017). Modelling non-stationary ion transfer in neutralization dialysis. J. Membr. Sci..

[40] Lide D.R. (2005). Handbook of Chemistry and Physics.

[41] Zabolotskii V.I., Loza S.A., Sharafan M.V. (2005). Physicochemical properties of profiled heterogeneous ion-exchange membranes. Russ. J. Electrochem..

[42] Berezina N.P., Kononenko N.A., Dyomina O.A., Gnusin N.P. (2008). Characterization of ion-exchange membrane materials: Properties vs structure. Adv. Colloid Interfac..

[43] Vermaas D.A., Kunteng D., Saakes M., Nijmeijer K. (2013). Fouling in reverse electrodialysis under natural conditions. Water Res..

[44] Gnusin N.P., Karpenko L.V., Demina O.A., Berezina N.P. (2001). Calculation of the ion-exchange equilibrium constant for MK-40 sulfo cation-exchange membranes from conductometric data. Rus. J. Phys. Chem..

[45] Kozmai A.E., Nikonenko V.V., Zyryanova S., Pismenskaya N.D., Dammak L., Baklouti L. (2019). Modelling of anion-exchange membrane transport properties with taking into account the change in exchange capacity and swelling when varying bathing solution concentration and pH. J. Membr. Sci..

[46] Sousa P., Soares A., Monteiro E., Roubo A. (2014). A CFD study of the hydrodynamics in a desalination membrane filled with spacers. Desalination.

